# Professional practice changes in radiotherapy physics during the COVID-19 pandemic

**DOI:** 10.1016/j.phro.2021.06.002

**Published:** 2021-06-22

**Authors:** Jenny Bertholet, Marianne C. Aznar, Cristina Garibaldi, David Thwaites, Eduard Gershkevitsh, Daniela Thorwarth, Dirk Verellen, Ben Heijmen, Coen Hurkmans, Ludvig Muren, Kathrine Røe Redalen, Frank-André Siebert, Marco Schwarz, Wouter Van Elmpt, Dietmar Georg, Nuria Jornet, Catharine H. Clark

**Affiliations:** a European Society for Radiotherapy and Oncology (ESTRO), Physics Committee, Brussels, Belgium; b Division of Medical Radiation Physics, Department of Radiation Oncology, Inselspital, Bern University Hospital, University of Bern, Switzerland; c Division of Cancer Sciences, Faculty of Biology, Medicine and Health, The University of Manchester, The Christie NHS Foundation Trust, Manchester, UK; d Nuffield Department of Population Health, University of Oxford, Oxford, UK; e Unit of Radiation Research, IEO European Institute of Oncology, IRCCS, Milano, Italy; f Institute of Medical Physics, School of Physics, University of Sydney, Sydney, Australia; g Medical Physics, Leeds Institute of Cancer and Pathology, School of Medicine, Leeds University, Leeds, UK; h North Estonia Medical Centre, Tallinn, Estonia; i Section for Biomedical Physics, Department of Radiation Oncology, University Hospital Tübingen, Germany; j Iridium Network, Antwerp University (Faculty of Medicine and Health Sciences), Antwerp, Belgium; k Department of Radiotherapy, Erasmus MC Cancer Institute, University Medical Center Rotterdam, The Netherlands; l Catharina Hospital, Department of Radiation Oncology, Eindhoven, The Netherlands; m Danish Centre for Particle Therapy, Aarhus University Hospital, Aarhus, Denmark; n Department of Physics, Norwegian University of Science and Technology, Trondheim, Norway; o Clinic of Radiotherapy, University Hospital of Schleswig-Holstein, Campus Kiel, Germany; p Proton Therapy Department, Trento Hospital, TIFPA-INFN, Trento, Italy; q Department of Radiation Oncology (MAASTRO), GROW – School for Oncology, Maastricht University Medical Centre, Maastricht, The Netherlands; r Division Medical Radiation Physics, Department of Radiation Oncology, Medical University of Vienna, AKH Wien, Austria; s Servei de Radiofísica i Radioprotecció, Hospital de la Santa Creu i Sant Pau, Barcelona, Spain; t Department of Radiotherapy Physics, University College London Hospital, UK; u Department of Medical Physics and Bioengineering, University College London, UK; v National Physical Laboratory, London, UK

**Keywords:** COVID-19, SARS-CoV-2, Radiotherapy, Medical Physics, Treatment planning, Quality assurance

## Abstract

**Background and purpose:**

The COVID-19 pandemic has imposed changes in radiotherapy (RT) departments worldwide. Medical physicists (MPs) are key healthcare professionals in maintaining safe and effective RT. This study reports on MPs experience during the first pandemic peak and explores the consequences on their work.

**Methods:**

A 39-question survey on changes in departmental and clinical practice and on the impact for the future was sent to the global MP community. A total of 433 responses were analysed by professional role and by country clustered on the daily infection numbers.

**Results:**

The impact of COVID-19 was bigger in countries with high daily infection rate. The majority of MPs worked in alternation at home/on-site. Among practice changes, implementation and/or increased use of hypofractionation was the most common (47% of the respondents). Sixteen percent of respondents modified patient-specific quality assurance (QA), 21% reduced machine QA, and 25% moved machine QA to weekends/evenings. The perception of trust in leadership and team unity was reversed between management MPs (towards increased trust and unity) and clinical MPs (towards a decrease). Changes such as home-working and increased use of hypofractionation were welcomed. However, some MPs were concerned about pressure to keep negative changes (e.g. weekend work).

**Conclusion:**

COVID-19 affected MPs through changes in practice and QA procedures but also in terms of trust in leadership and team unity. Some changes were welcomed but others caused worries for the future. This report forms the basis, from a medical physics perspective, to evaluate long-lasting changes within a multi-disciplinary setting.

## Highlights

• The experience of 433 medical physicists during COVID-19 was analysed.
• Changes in clinical practice had an impact on treatment planning and quality assurance tasks.
• The effects of the changes were perceived differently by management vs clinical medical physicists.
• There is a clear willingness to learn from this experience.

## 1 Introduction

The outbreak of the Severe Acute Respiratory Syndrome CoronaVirus 2 (SARS-CoV-2) causing Coronavirus disease 2019 (COVID-19) is an extreme event with complex spread-dynamics [1]. The crisis has produced different infection rates and strain levels on the healthcare systems of different countries. Radiotherapy (RT) has been recognized as an essential non-elective treatment for many tumour entities [2], [3]. Cancer patients are at increased risk of COVID-19-related complications [4]. Although RT services continued, important changes were implemented rapidly to enhance patient and staff protection against infection, including treatment delay or RT schedule alterations [2], [3], [5], [6], [7], [8], [9], [10], [11], [12]. This also affected medical physicists (MPs), who are key healthcare professionals in sustaining safe, effective, and efficient RT under constraints of social distancing and national/regional pandemic-related guidelines [13].

In addition to legal requirements for MPs presence at the hospital, which may differ internationally, Whitaker et al. distinguished four types of tasks from a practical standpoint [14]. First, direct patient-facing tasks, such as brachytherapy, first fraction verification for complex treatments and *in-vivo* dosimetry represent a small but vital portion of the workload requiring MPs on-site during treatment hours. Second, some measurement-based quality assurance (QA) requires on-site presence but can be performed out of treatment hours. Third, treatment planning-related, quality management, and administrative tasks can often be done remotely. Fourth, project planning, education, training and research can often be performed remotely or potentially postponed for clinical staff.

Researchers that mostly have type-4 tasks were most likely to work from home while clinical MPs needed to work on-site and/or on shift [13], [14], [15]. Management MPs were involved in decision making and liaising between hospital administration and clinical staff [9]. Thus, MPs may have had widely diverse experiences during COVID-19 due to different roles and responsibilities.

Most RT service changes were aimed at limiting patient presence at the hospital (e.g. RT postponement when clinically supported, or hypofractionation) and to limit interaction between team-members (e.g. shift work) [2], [5], [6], [7], [8], [9], [10], [16], [17], [18]. These were considered a “contingency standard of care” in an “early pandemic scenario” while more drastic resource shortages and patient triage were anticipated in a “second (later) pandemic scenario” [19], [20]. MPs have less patient contact than radiation oncologists, radiation therapists (RTTs) and nurses. They perform many “behind the scenes” tasks, e.g. planning and patient-specific QA (PSQA), easily overlooked when establishing guidelines limiting patient contact. Whereas new publications are exploring the aftermath and lessons learned for future clinical practice [18], [21], [22], [23] with potentially more patient-centric research and streamlined administrative and regulatory procedures [24], the impact for MPs is unclear.

This study presents a global survey of MPs experience during the first pandemic wave (March-June 2020). We aim to understand the different challenges faced by MPs in different roles and countries and the potential impact for future practice. Results were analysed for three country clusters based on daily number of infections in order to link the spread-dynamic and early/late pandemic scenarios to changes in medical physics practice. Results are also reported by professional role (management vs clinical MP) where relevant. From this study we can infer how to adapt to future crises, but also integrate potentially positive changes to keep for the future.

## 2 Materials and methods

A web-based questionnaire (Supplementary material A.I) was distributed to ESTRO’s physics members via e-mail (2500 recipients) and the broader medical physics community via social media. It contained 39 questions on demographics (questions 1–12), department organisation (13–20), changes in practice (21–31), morale and mental health (32–33) and expected future impacts (34–38), with an open text-box for further comment(s). Responses were collected anonymously from June 18 to September 24, 2020.

Of the 489 responses received, 50 with no information beyond the demographics and 6 from non-MP professionals were excluded. Results from the 433 respondents were analysed overall, as well as by professional group and country cluster, where relevant.

The largest professional groups were clinical MPs (N = 298, 69%) and head of medical physics/management (hereafter “management MPs”, N = 110, 25%). Fifteen research/academic physicists (3%) and 10 with other or non-specified roles (2%) were only included in the overall analysis.

Countries were divided into clusters according to the daily number of infections [1], with cluster A (N = 222, 51%) containing Italy, Spain, UK, USA, that were generally most affected by the pandemic. Cluster B (N = 156, 36%) contained countries such as Belgium, the Netherlands, Switzerland. Cluster C (N = 45, 10%) contained countries with low daily case numbers, with most responses from Norway and New Zealand (see supplementary material A.II for details). Ten responses were not included in any cluster, but only in the overall analysis, because either country was not indicated or was not included in the analysis of [1]. The majority (68%) of responses came from Europe followed by US/Canada (16%), New Zealand and Australia (7%), Asia and Middle East (7%), Latin America and Africa (~1% each).

Demographics for clusters A and B were homogeneous (Supplementary Table A.III).

No response was mandatory and the number of responses per country were uneven. Therefore, no statistical analysis between sub-groups could be performed.

## 3 Results

### 3.1 Organisation of the department

(Cluster) A-respondents were the most tested for COVID-19, and had the highest rates of positive/suspected patients and related treatment interruption (Table 1). The corresponding test rates and positive test rates were lower for B and lowest for C-respondents. A-respondents were more likely to be tested for COVID-19 after the first crisis peak, while B-respondents were more often tested before or at the crisis peak.

**Table 1:** Infection situation in the department by country cluster and overall.

By cluster^a^	A (N = 222)	B (N = 156)	C (N = 45)	Overall (N = 433)
**Were you tested for COVID-19 (e.g. blood, nose/mouth swab)? (Q9/10)**				
No	104 (47%)	88 (56%)	36 (80%)	234 (54%)
Yes, at the start of the crisis	11 (5%)	24 (15%)	4 (9%)	40 (9%)
Yes, at the peak b of the crisis	29 (13%)	27 (17%)	3 (7%)	62 (14%)
Yes, after the peak of the crisis	78 (35%)	17 (11%)	2 (4%)	97 (22%)

**Was any patient COVID-positive (or suspected) treated in your department? (Q11)**				
Yes	152 (68%)	85 (54%)	11 (24%)	251 (58%)
No	47 (21%)	51 (33%)	23 (51%)	127 (29%)
I don’t know	22 (10%)	20 (13%)	11 (24%)	54 (12%)
No response	1	0	0	1 (<1%)

**Did any patient have their treatment interrupted because of confirmed/suspected COVID-19 infection? (Q12)**				
Yes	99 (45%)	57 (37%)	13 (29%)	172 (40%)
No	75 (34%)	61 (39%)	19 (42%)	162 (37%)
I don’t know	47 (21%)	38 (24%)	13 (29%)	98 (23%)
No response	1	0	0	1 (<1%)

a Ten responses are not associated with any cluster (see supplementary material A.II).

b this question referred to the first peak of the crisis (March–June 2020).

The proportion of respondents working at home or on location was similar across clusters (Supplementary Fig. 1). Only a third of clinical MPs worked fully on-site.

Thirty percent of A-respondents reported that there was no contingency plan to handle the COVID-19 situation while for B and C-respondents this was 10% and 16%, respectively (Table 2).

**Table 2:** Organisation of the department by country cluster and overall.

By cluster^a^	A (N = 222)	B (N = 156)	C (N = 45)	Overall (N = 433)
**How well prepared was the department for the COVID-19 emergency? (Q13)**				
Some contingency plan, but we had to develop the plan further as we went along	132 (59%)	93 (60%)	21 (47%)	253 (58%)
Well-developed contingency plan	44 (20%)	46 (29%)	13 (29%)	105 (24%)
No contingency plan	44 (30%)	15 (10%)	7 (16%)	67 (15%)
Other	1^b^	2 (1%)^c^	1 (2%)^d^	4 (<1%)
No response	1	0	3 (7%)	4 (<1%)

**Did you divide the team? (Q14/15)**				
Yes split but not alternate (one group at home)	23 (10%)	21 (13%)	4 (9%)	52 (12%)
Yes split and alternate between work/home	117 (53%)	72 (46%)	34 (76%)	227 (52%)
No, no split	56 (25%)	38 (24%)	4 (9%)	100 (23%)
Other (see text for description)	26 (12%)	24 (15%)	3 (7%)	53 (12%)
No response	0	1 (<1%)	0	1 (<1%)

**Did you divide the department into clean/at risk areas? (Q16)**				
Yes	79 (36%)	55 (35%)	12 (27%)	148 (34%)
No	130 (59%)	85 (54%)	27 (60%)	249 (57%)
Other (see text for description)	11 (5%)	13 (8%)	4 (9%)	29 (7%)
No response	2 (<1%)	3 (2%)	2 (4%)	7 (2%)

**Did you have the means to work remotely in planning? (Q17)**				
No, we did not get remote connection	30 (14%)	45 (29%)	13 (29%)	92 (21%)
Yes, we got it as soon the emergency started	48 (22%)	21 (13%)	6 (13%)	76 (17%)
Yes, we already had it	90 (41%)	63 (40%)	22 (49%)	180 (41%)
Yes, we got it but not immediately	43 (19%)	16 (10%)	4 (9%)	63 (14%)
Other	9 (4%)	10 (6%)	0	19 (4%)
No response	2 (<1%)	1 (<1%)	0	3 (<1%)

**Was the physics personnel screened daily for COVID symptoms? (Q18)**				
Yes	84 (38%)	85 (54%)	12 (27%)	185 (43%)
No	138 (62%)	70 (45%)	29 (64%)	247 (57%)
No response	0	1 (<1%)	4 (9%)	1 (<1%)

**Which personal protective equipment was available from the hospital for MPs at the peak of the crisis? (Q19)**				
Gloves	131 (59%)	85 (54%)	30 (67%)	254 (58%)
FFP2/N95 mask	44 (20%)	38 (24%)	6 (13%)	92 (21%)
Surgical mask	190 (86%)	101 (65%)	28 (62%)	329 (76%)
Protective glasses	29 (13%)	23 (15%)	4 (9%)	59 (14%)
Visor	23 (10%)	25 (16%)	9 (20%)	60 (14%)
Other (see text)	38 (17%)	34 (22%)	3 (7%)	62 (14%)

**What proportion of your physics staff had to stay away from the department for COVID-related reasons? (Q20)**				
Due to being infected themselves				
None	129 (58%)	104 (67%)	34 (76%)	275 (63%)
<10%	42 (19%)	21 (13%)	3 (7%)	66 (15%)
10–25%	19 (9%)	9 (6%)	1 (2%)	29 (7%)
25–50%	6 (3%)	5 (3%)	0	11 (3%)
>50%	0	3 (2%)	0	3 (<1%)
No response/do not know	26 (12%)	14 (9%)	7(16%)	49 (12%)

Due to going into isolation (e.g. household member infected/infection suspected)				
None	99 (45%)	82 (53%)	22 (49%)	210 (48%)
<10%	60 (27%)	30 (19%)	11 (24%)	101 (23%)
10–25%	33 (15%)	22 (14%)	5 (11%)	60 (14%)
25–50%	9 (4%)	6 (4%)	1 (2%)	17 (4%)
>50%	1 (<1%)	3 (2%)	2 (4%)	6 (1%)
No response/do not know	20 (90%)	13 (8%)	4 (9%)	39 (9%)

a Ten responses are not associated with any cluster (see supplementary material A.II).

b Describes a quick adaption to COVID-19.

c One had no plan initially, then developing their approach then hospital-wide plan in place. One had a plan before the emergency started.

d Well-developed department plan but hospital-wide plan lagged behind.

Three-quarters of respondents indicated some form of team splitting, mostly implemented by alternating people at home or at work with daily, weekly, or less frequently, early/late shifts. For small departments with 1–2 treatment units, 30% of respondents indicated there was no team splitting. This number was 23%, 16% and 21% for departments with 3–6 units, 7–10 units or 10 or more units respectively. Some other arrangements, aimed at minimizing contact at work, included groups on-site without contact or people working at home whenever possible.

For just over half of the respondents, departments were not split into at risk/clean areas (Table 2). However, about a third of respondents’ departments, relatively homogeneous across clusters, were split. “Other” solutions involved having one COVID-linac or treating at-risk patients at the end of the day.

Although remote access for planning was similar across the clusters (40%) before the pandemic, access increased more for A-respondents than for B and C-respondents during the pandemic.

Access to personal protective equipment (PPE) was similar across clusters (Table 2). Yet 13 A-respondents and eight B-respondents reported explicitly having no, or insufficient, equipment. Eight B-respondents reported no such need because contact could be avoided. Some reported having specialized equipment (visor, high filtration masks) for brachytherapy.

The percentage of physics staff staying away due to being infected was highest in cluster A and lowest in C (Table 2). The percentage staying away due to quarantine was higher than for infection, and cluster A had the highest rates. Other common reasons to stay away were risk factors or pregnancy (self or relative, 49 respondents), child-care (13), suspected infection/awaiting test results (8) or travel (self or relative, 6). Two respondents reported that staff remained at home because of pandemic-related anxiety or distress.

### 3.2 Changes in practice

**Fig. 1:**
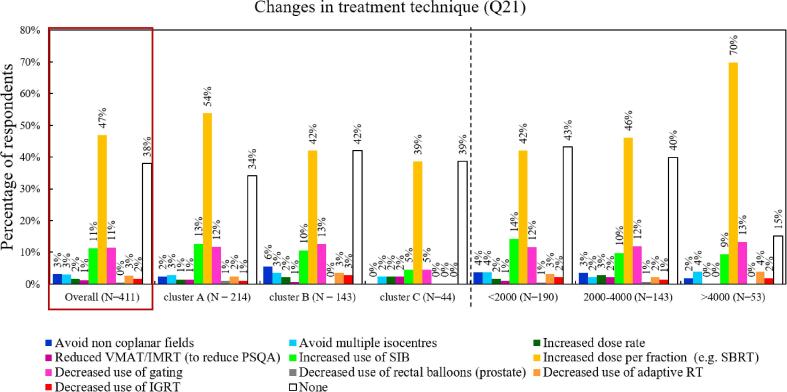
Changes in treatment technique (Q21) overall (red box), by country cluster (left of the dotted line) and by centre size in patients treated per year (right of the dotted line). Ten responses not associated to any cluster and 25 responses without an answer for the number of patients treated per year are only included in the “Overall” group. (For interpretation of the references to colour in this figure legend, the reader is referred to the web version of this article.)

This section is based on 411 responses. The most widely implemented change was increased use or implementation of hypofractionation (193 responses, 47%, Fig. 1). This was more prevalent in cluster A (54%) and in large-volume centres (4000+patients/year, 70%). Decreased gating use and increased simultaneous integrated boost (SIB) use was reported by 11% of respondents. Overall, 156 (38%) respondents implemented no changes in techniques (15% for large-volume centres).

In addition, 16 respondents observed a reduction in RT and 11 mentioned postponing prostate RT with longer hormone treatment instead. Fifty-six (14%) respondents (37 from cluster A) observed a workload increase, including 13 where RT was given instead of surgery and/or chemotherapy. Six had more head and neck referrals; five had additional data collection or reporting; and four reported increased workloads due to initial replanning or additional QA for hypofractionated treatments.

PSQA and machine QA procedures were changed/reduced by 16% and 21% of respondents respectively (Table 3). For 25%, QA was moved to weekend or evenings to limit interactions. Others often did QA “whenever possible”.

**Table 3:** Changes in QA practice and time required to sterilise the linac room by country cluster and overall.

By cluster^a^	A (N = 214)	B (N = 143)	C (N = 44)	Overall (N = 411)
**Did you change patient specific QA? (Q22)**				
No, we continued the same way	181 (85%)	121 (85%)	37 (84%)	346 (84%)
Reduction of pre-treatment QA	15 (7%)	4 (3%)	2 (5%)	23 (6%)
Reduction or stopped in-vivo dosimetry (diodes)	7 (3%)	3 (2%)	0	10 (2%)
Increase use of EPID or other online in-vivo QA	7 (3%)	5 (3%)	1	15 (4%)
Increase use of remote automatic PSQA	5 (2%)	2 (1%)	1	8 (2%)
Other	13 (6%)	8 (6%)	3 (7%)	24 (6%)

**Did you change the tests for treatment unit QA? (Q23/24)**				
No, we did not change	176 (82%)	111 (78%)	33 (75%)	326 (79%)
Yes, we stopped yearly/quarterly tests	12 (6%)	6 (4%)	4 (9%)	23 (6%)
Yes, we reduced tests frequency	14 (7%)	12 (8%)	4 (9%)	32 (8%)
Yes, we reduced the number of tests	14 (7%)	6 (4%)	0	22 (5%)

**Did you change the time for treatment unit QA? (Q25)**				
We kept the same machine QA slots	135 (68%)	99 (69%)	30 (68%)	269 (65%)
We moved QA to a different slot	59 (28%)	30 (21%)	10 (23%)	103 (25%)
Other	14 (7%)	4 (3%)	1 (2%)	20 (5%)
No response	6 (3%)	10 (7%)	3 (7%)	19 (5%)

**To compensate for the extra time needed to sterilise the linac room, after treating a COVID patient, RT/MP services needed to (Q29):**				
Extend working hours	28 (13%)	34 (24%)	4 (9%)	68 (17%)
Reduce the number of patients treated	45 (21%)	18 (13%)	7 (16%)	72 (18%)
No changes	99 (46%)	73 (51%)	26 (59%)	203 (49%)
Other (see text)	42 (20%)	15 (10%)	5 (11%)	62 (15)

Acronyms: EPID: electronic portal imaging device.

a 10 responses are not associated with any cluster (see supplementary material A.II).

Overall, 49% of respondents did not need changes to compensate for extra time for cleaning/sterilizing rooms after treating COVID-positive patients, while 17% and 18% extended working hours and reduced patient numbers, respectively (Table 3). Thirteen respondents indicated that no COVID-positive/suspected patients had been treated in their department. Twelve reported that the reduced patient numbers compensated for the extra cleaning time.

Most respondents reported that treatment unit technical support was available (Supplementary Table A.IV). Preventive maintenance was cancelled for 30% of A-respondents, 22% of B and 27% of C. Replacements of the high-dose rate/pulsed-dose rate afterloader sources were generally carried out as planned.

Some MPs participated in COVID-related research initiatives: 49 respondents reported that MPs helped design databases, 48 collected data (e.g. cone-beam CT), 15 participated in radiomics studies, eight investigated low-dose RT as COVID-19 treatment, four wrote papers on their experience and one investigated mask sterilization.

### 3.3 Morale and mental health

This section is based on 410 responses. To keep the team united during the pandemic, 282 (69%) respondents used email, 199 (49%) texting groups, 108 (26%) voice-only teleconference, 195 (48%) video-conferencing, 143 (35%) communication and collaboration platforms, and 124 (30%) on-site face-to-face meetings.

Additionally, 20 respondents reported virtual social interactions. Fifteen respondents reported that staff had supported each other (as a personal initiative). Four wrote that the lack of support had been hard on them, while seven indicated there was no need for any support. Eight people mentioned increased flexibility in working time, six that there was some kind of support, and six mentioned psychological support available at various levels.

### 3.4 Future impact

**Fig. 2:**
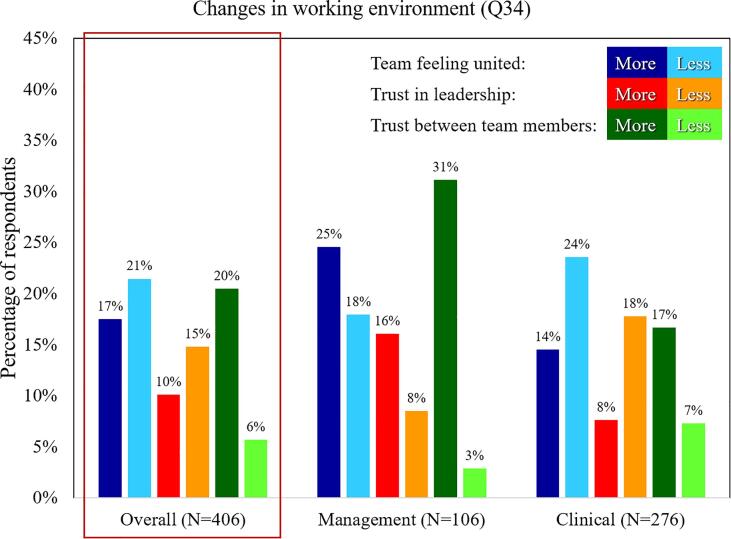
Changes observed in working environment by professional group and overall. For a same colour, darker shades indicate an increase in unity/trust whereas a lighter shade indicates a decrease.

This section is based on 406 responses. Over all professional groups, more respondents noted an increase, rather than a decrease, in trust between team members (Fig. 2). For management MPs, responses indicated an increased feeling of team unity and trust in leadership. However, for clinical MPs, the tendency was reversed.

**Fig. 3:**
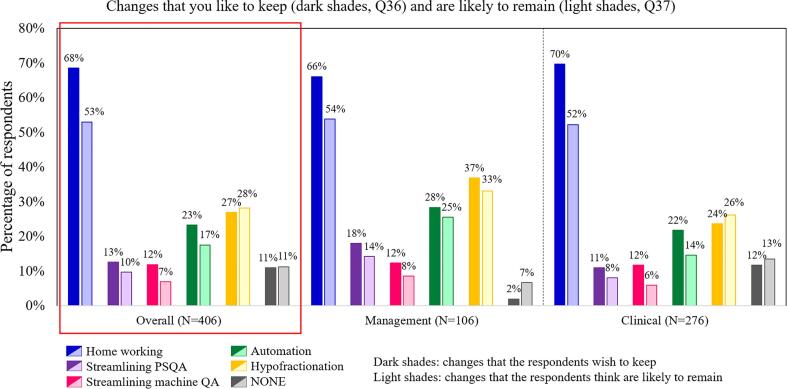
Changes in practice for the future that respondents wish to keep (in dark shades) and how likely they are to remain (light shades). Results are presented overall in the red box and for management and clinical MPs on the left and right of the dotted line respectively. (For interpretation of the references to colour in this figure legend, the reader is referred to the web version of this article.)

Over 70% of respondents, over all professional groups, reported that working from home had become more acceptable and 48% reported that flexible working had become more acceptable. Forty percent of respondents reported the use of new online tools. Sixty-eight percent of respondents would like home-working to remain after the pandemic and a slightly lower number believes that is likely (Fig. 3). Regarding clinical practice, there is some interest in keeping more hypofractionation after the pandemic and a similar number said that is likely. Generally, wishes and likeliness for changes was evaluated slightly higher by management MPs than by clinical MPs. Some respondents noted other things they would like to remain: paperless clinics (9), online meetings (6), flexible working (5) and more online conferences or exams (5). Few respondents (45 overall, 11%) hope that no changes will remain after the pandemic.

The majority of respondents (83%) were not concerned about pressure to keep changes made to cope with increased workload, but 48 (12%) had concerns (14% of management, 12% of clinical MPs). Among these, 21 mentioned pressure for out-of-hours work (week-end, evenings). Seven were concerned about increased workloads or less time/staff for the same amount of work.

Finally, other comments shared at the end of the survey generally indicated a willingness to learn and reflect on the changes necessitated by COVID-19. Some wrote that they were proud of their team and management and of how they had been well prepared. In contrast, others were disappointed in management and/or felt unheard. Unedited, anonymous comments are available in supplementary material A.IV.

## 4 Discussion

We report on the experience of 433 MPs from 40 countries worldwide during the early stage of the COVID-19 pandemic. Overall, they observed substantial changes in working situations and their institution’s clinical practice. Shift working with weekly alternation at-home/on-site with minimal contact and the use of PPE was frequently adopted to limit the infection risk for clinical MPs.

Countries were grouped in clusters based on COVID-19 data up to April 12, 2020 [1] while responses were collected between June-September 2020. The cluster-based analysis may be biased with certain countries or centres being over-represented. Moreover, because the situation is highly dynamic, this survey should be considered a snapshot in time. Different pandemic stages have been identified as “early pandemic scenario” aiming for contingency standard of care and “late pandemic scenario” anticipating more drastic resource shortages and patient triage [19], [20]. A-countries had typically reached the latter stage early, while C-countries mostly remained in the early stage at the survey time and could learn from A-countries experience. This difference in burden on healthcare systems was reflected in the department infection situation and organisation (Table 1, Table 2). A-respondents had the highest numbers of infected staff or those staying away from the department and the most infected/suspected patients. They were also most tested for COVID-19 but typically after the first peak, likely due to shortage of tests early in the pandemic, e.g. as reported in Spain [9]. Conversely, B-respondents were more often tested at the crisis start or peak (Table 1). Access to PPE at the first crisis peak for A-respondents was similar to other clusters. However, thirteen A-respondents reported having no or insufficient hospital-provided PPE, consistent with reported PPE shortages [2], [3], while some B-respondents reported not needing PPE because contact could be avoided. Having little patient contact, MPs might have had low priority for PPE access despite the risk of infection among colleagues.

There was little difference in change of practice between clusters (Fig. 1, Table 3). Nevertheless, the most common change, implementation or increased use of hypofractionation (47% overall), was more predominant in cluster A (54%). The increase was markedly more common in centres with large patient numbers and it has been proposed extensively to reduce the patient presence in the hospital [12]. However, hypofractionation use is country-dependent [17], [25] and for centres not yet applying it, implementation represents significant work, not easy to carry out under pressure [12]. Concerns around its level of evidence have also been raised which may have prevented wider implementation [12], [20]. Despite its advantages, hypofractionation may involve increased MPs workload, from PSQA, first fraction verification, and initial replanning at implementation. Others reported that already planned hypofractionation implementation or paperless clinics, were accelerated by the pandemic.

Certain medical physics tasks can, in principle, be performed remotely (e.g. treatment planning) or on-site outside of treatment hours (e.g. machine QA) [14]. Indeed, remote planning access was already available before COVID-19 for about 40% of respondents. However, 14% of A-respondents and 29% of B and C-respondents had not yet got remote planning access during COVID-19. Khan et al. claim that the bulk of planning and plan checking could be performed remotely without quality loss [13], even if not supported by data. Although technically feasible, it has a cost and may be complicated by cyber-security and data-privacy perspectives [26], [27]. Adequate equipment (e.g. computer hardware, fast internet connection) may be required for efficient and comfortable home-working for MPs. A full health and safety assessment for home-working environments should be considered, as Whitaker et al. recommend [14], if it becomes routine practice.

Concerning on-site QA tasks, 16% and 21% of respondents changed PSQA and reduced machine QA, respectively (Table 3). A quarter of respondents moved QA to different times, often weekend or evening. These changes align with Khan et al. who reported prioritisation of QA tasks and moving QA to evenings/weekends or early morning before first patient [13]. However, it is necessary to establish clear guidelines prioritising QA tasks/test frequency in the case of resource shortage. Within the interdisciplinary team, the workload involved in hypofractionation implementation and PSQA should be accounted for when recommending regimen alteration, especially in emergency situations.

Multiple reports have warned of expected increased workloads post-pandemic from deferred referrals and patient treatments [9], [13], [28], [29]. For MPs, postponed QA and equipment servicing may also contribute to substantial workload increases [13]. Among respondents, there was clear interest in increasing working flexibility and home-working which may help in handling this backlog [13]. Although some changes such as the implementation of hypofractionation and home-working were welcomed, personal sacrifices were also reported. Twelve percent of respondents were concerned about pressure to maintain increased or out-of-hours workloads. Management MPs were as concerned as clinical MPs, indicating that this pressure may come from higher in the department or hospital management.

Interestingly, respondents indicated overall that there had been increased trust between team members (Fig. 2), but the tendencies regarding trust in leadership and team unity were reversed between management MPs (towards increased trust and unity) and clinical MPs (towards a decrease). This contradiction is concerning given that the perception of being valued by the supervisor was related to decreased burnout-risk among MPs [30] and trust in leadership rests on a fragile balance of mutual trust and transparent communication [31], [32]. While some respondents indicated specific actions to keep the team united and maintain social interaction, some also reported suffering from the situation. Although virtual social interactions increased over the past year, some people might remain isolated with a risk of increased anxiety and depression symptoms [15]. Reaching out to colleague to keep social interaction may be an easy but effective way to maintain moral.

One main limitation of the study is the sparsity of the data: a response was not mandatory for any of the questions (to encourage a high response rate); a small number of responses were received from research MPs; and the number of responses per country was uneven with most responses coming from Europe. The second main limitation is that the data was collected in late summer 2020 in a highly dynamic situation. Nevertheless, our results indicate possible underlying causes and mechanisms of medical physics practice adaptation. The changes deployed to deal with the pandemic have highlighted that certain tasks can sometimes be performed more efficiently, from one or more, or combinations of, process and work alterations, (e.g. remote working, use of online systems, working pattern modifications). Some are viewed positively by staff, but others have the potential to add stress in the longer term or be more difficult to manage and sustain and so need careful discussion and be acceptable to the staff involved. As of February 2021, a majority of staff in Europe had received their first vaccination dose but 76% of department heads were concerned about their employees’ mental health, risk of burnout and well-being at work, acknowledging the effect of the pandemic on workload and work-life balance [33]. A year on, remote training and (continuous) education have also been widely implemented [34]. As live or blended courses and congresses are being offered again [35], the opportunity for broader access in the virtual world should be exploited. The increased use of automation and hypofractionation will likely be accelerated by COVID-19 and guidelines on the prioritization of QA tasks in case of future resource shortage are expected in the short term but should be based on international consensus.

The challenge, looking ahead post-pandemic, is to optimize resource-use while maintaining treatment quality and safety and ensure an equitable workload and sustainable working conditions for all professions. This task should include all stakeholders and further qualitative assessment via focus groups and/or new surveys to capture the evolution of clinical practice and COVID-19′s long-lasting effects. As previous reports considered the lessons learned from the oncologists [24] or RTTs [18] perspective, we hope that this report can constitute a basis to start this multi-disciplinary assessment from the medical physics perspective.

In conclusion, MPs have reported a large impact of COVID-19 on their work, depending on the number of daily infections in their country. This caused changes in practice such as increased use/implementation of hypofractionation and reorganisation of machine and patient-specific QA. The impact of these changes on team unity and trust in leadership has been perceived differently by management and clinical MPs. Although some changes were welcomed, there were also personal sacrifices. We hope this data will help reflect on the impact of this evolution on MPs for future crisis scenarios but also in regular practice.

## Declaration of Competing Interest

The authors declare that they have no known competing financial interests or personal relationships that could have appeared to influence the work reported in this paper.

## Appendix A Supplementary data

Supplementary Figure 1 Working situation overall (red box), by country cluster (left of the dotted line) and by professional group (right of the dotted line).

Supplementary Material A.I Web-based questionnaire in PDF.

Supplementary Material A.II Country Clustering.

Supplementary Material A.III Demographics by country cluster and overall.

Supplementary Material A.IV Technical support availability and machine maintenance by country cluster and overall.

Supplementary Material A.V Free text comments from the respondents.
