# Supplementary figures and images for: Professional practice changes in radiotherapy physics during the COVID-19 pandemic
Source: Phys Imaging Radiat Oncol. 2021 Jun 22;19:25–32. doi: 10.1016/j.phro.2021.06.002 (PMC8216850; doi:10.1016/j.phro.2021.06.002)

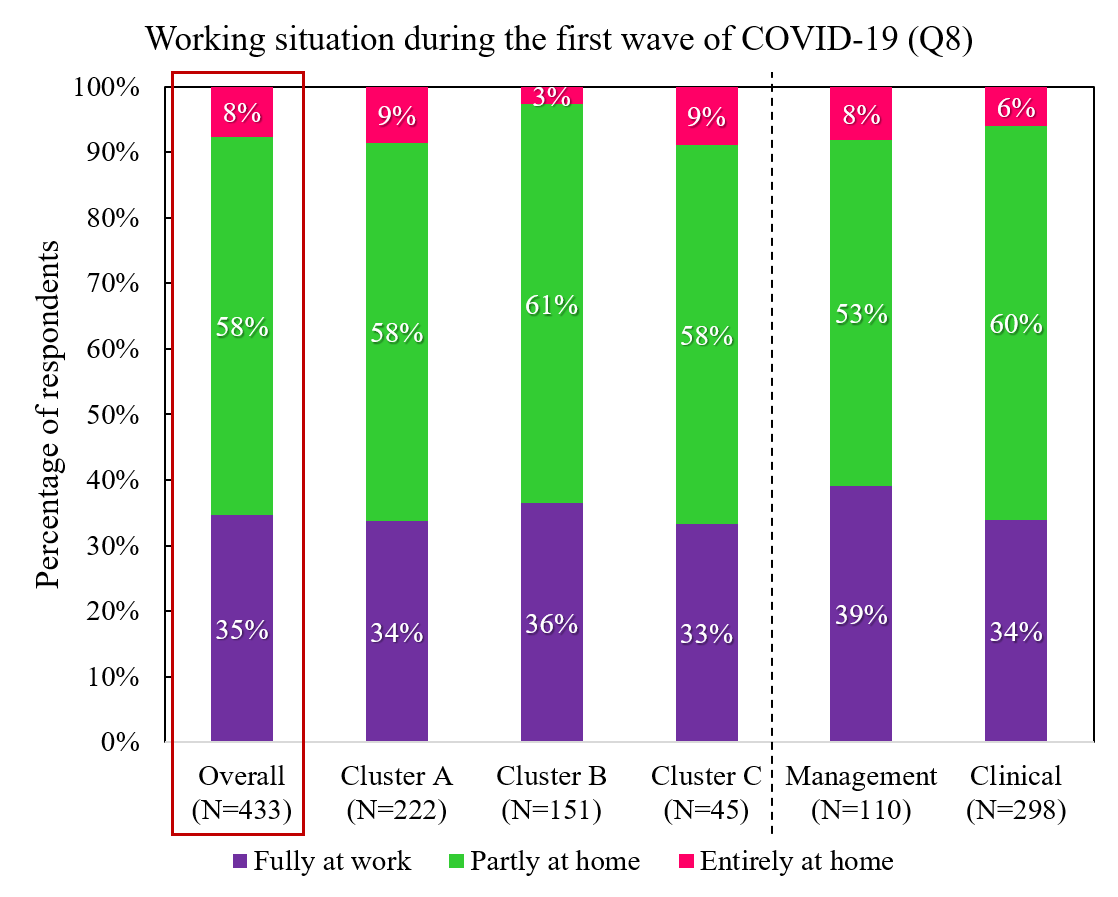

Supplement: Supplementary Figure 1 — Working situation overall (red box), by country cluster (left of the dotted line) and by professional group (right of the dotted line). [file mmc1.zip › SuppFig1.png]
