# Supplementary material for: Professional practice changes in radiotherapy physics during the COVID-19 pandemic
Source: Phys Imaging Radiat Oncol. 2021 Jun 22;19:25–32. doi: 10.1016/j.phro.2021.06.002 (PMC8216850; doi:10.1016/j.phro.2021.06.002)
Supplement: Supplementary Material A.I — Web-based questionnaire in PDF. [file mmc2.pdf]

Lessons learned in radiotherapy physics during the Covid-19 pandemic

Welcome

This survey has been developed by the Physics Committee of ESTRO. The aim is to gather the lessons learned in radiotherapy physics during the Covid-19 pandemic of 2020. The results will be used for a symposium at ESTRO 2021 in Madrid.

We want to gather all experiences (good and poorer) from all physicists working in radiation oncology, so multiple physicists can answer from the same institution.

Results will be anonymous (including city and centre info) It is helpful if you can answer the questions as fully as possible, however no question is mandatory. The questions have been divided into sections on demographics, organisation of the department, changes in practice, morale and mental health and impact for the future. There are 35 questions in total and most are tick box responses.

Thank you for participating in this survey.

Lessons learned in radiotherapy physics during the Covid-19 pandemic

Demographics

Questions about your institution and yourself

1. What is your age?

- ◯ 18-24
- ◯ 25-34
- ◯ 35-44
- ◯ 45-54
- ◯ 55-64
- ◯ 65+

2. To which gender identity do you most identify?

- ◯ Female
- ◯ Male
- ◯ Non-binary
- ◯ Prefer not to say

3. In which country do you work?

4. In which city do you work?

5. What is your position?

6. Number of treatment units (linacs , brachy afterloader, proton units) in your department

- 1-2
- 3-6
- 7-10
- 10+

7. Number of patients per year

- < 2000
- 2000-4000
- 4000+

8. Were you working from home during the Covid pandemic?

- Yes, entirely
- Yes, partly
- No

9. Were you tested (e.g. using blood / nose, mouth swab) for Covid-19 at any time?

- Yes
- No

10. If yes – when was the first test done (approximately)?

- at the start of the crisis
- at the peak of the crisis
- after the peak of the crisis

11. Was any patient considered Covid positive (or Covid-suspected) treated in your radiotherapy department?

- Yes
- No
- I don't know

12. did any patient have their treatment interrupted because of confirmed / suspected Covid infection?

- Yes
- No
- I don't know

Lessons learned in radiotherapy physics during the Covid-19 pandemic

Organisation of the department

13. How well prepared was the department for the Covid-19 emergency (did you have a plan having seen the situation in other countries?)

- ◯ No contingency plan
- ◯ Some contingency plan, but we had to develop the plan further as we went along
- ◯ Well-developed contingency plan
- ◯ Other (please enter text)

14. Did you divide the team to have part of the team on site and part of the team working from home (in order to keep them safe and able to swap with the on-site team)?

- ◯ Yes, the team was split and alternated between home / work
- ◯ Yes, the team was split but did not alternate (i.e. a group stayed at home)
- ◯ No, the team did not split
- ◯ Other (please enter text)

15. If yes and alternation, what was the periodicity of alternation?

- daily
- 1 week
- 2 weeks
- Other (please enter text)

16. Did you divide the department into "clean" and "at risk/Covid+" areas?

- Yes
- No
- Other (please enter text)

17. Did you have the means to work remotely in planning?

- Yes, we already had remote connection to the hospital servers including TPS.
- Yes, we got remote connection including TPS as soon as the emergency started.
- Yes, we got remote connection including TPS, but not immediately
- No, we didn’t get remote connection
- Other (please enter text)

18. Were the physics personnel screened daily for any COVID symptoms or temperature before going into the department?

- ◯ Yes
- ◯ No

19. Which personal protective equipment was available from the hospital <u>for physicists</u> at the peak of the crisis? Tick availability

- ☐ Gloves
- ☐ FFP2/N95 mask
- ☐ Surgical mask
- ☐ Protective glasses
- ☐ Visor
- ☐ Other (please enter text)

20. What proportion of your physics staff had to stay away from the department for Covid-related reasons?

	None	less than 10%	10-25%	25%-50%	over 50%
Due to being infected themselves	○	○	○	○	○
Due to going into isolation (e.g. household member infected / infection suspected)	○	○	○	○	○
Other (please specify reason below)	○	○	○	○	○

If you answered "Other" please specify:

Lessons learned in radiotherapy physics during the Covid-19 pandemic

Change in practice

21. Did you change any treatment planning procedures? (Tick all relevant answers)

- ☐ Avoid non coplanar fields
- ☐ Avoid multiple isocentres
- ☐ Increased dose-rate
- ☐ Reduce VMAT, IMRT treatments to avoid pre-treatment QA
- ☐ Increase number of Simultaneous Integrated Boost treatments
- ☐ Increased dose per fraction (e.g. SABR/SBRT, 5 fractions for breast, etc...)
- ☐ Reduction of gated breathing techniques
- ☐ Reduction of the use of the rectal balloons for prostate treatments
- ☐ Decreased use of adaptive radiotherapy
- ☐ Decreased use of IGRT
- ☐ None
- ☐ Other (please enter text)

22. Did you change patient specific QA procedures? (Tick all relevant answers)

- [ ] No, we continued with the same patient specific QA
- [ ] Reduction of pre-treatment QA
- [ ] Reduction or stopped use of in vivo dosimetry using diodes
- [ ] Increased use of EPID or other online pre-treatment and in vivo QA
- [ ] Increased use of remote automatic patient specific QA
- [ ] Other (please enter text)

23. Did you change the tests for treatment unit QA?

- [ ] No, we didn’t change our machine QA
- [ ] yes, we reduced QA compared to our pre-Covid practice
- [ ] Other (please enter text)

24. if yes, which test did you change?

- [ ] stopped yearly / quarterly tests
- [ ] made tests less frequent
- [ ] reduced the number of tests
- [ ] we performed only daily QA
- [ ] Other (please specify)

25. Did you change the time for treatment unit QA?

- ◯ We kept the same machine QA time slots
- ◯ We moved the machine QA to a different time slot (eg night/weekends) to limit interactions
- ◯ Other (please enter text)

26. Was support from technical services for the treatment units available?

- ☐ Yes
- ☐ Yes, but only if the linac could not treat patients
- ☐ Yes, but only remote support
- ☐ No
- ☐ Other (please enter text)

27. Did you have preventive maintenance done on your treatment units during lockdown?

- ◯ No: it was not planned anyway
- ◯ No: it was planned but it was cancelled
- ◯ Yes: it was planned and carried out
- ◯ Other (please enter text)

28. Did you experience an unexpected additional workload e.g. increased referrals for RT instead of surgery (lung SBRT) or demands for data collection?

- ◯ No
- ◯ I don't know
- ◯ Yes (please describe)

29. To compensate for the extra time needed to sterilise the linac room, after treating a Covid patient, etc. Did RT/MP services need to:

- ☐ Extend the working hours?
- ☐ Reduce the number of patients treated?
- ☐ No change to RT/MP services
- ☐ Other (please enter text)

30. Did you have to change the HDR/PDR source in your afterloader?

- ◯ No, we don't have a HDR/PDR brachy afterloader
- ◯ No, we have a HDR/PDR afterloader but didn't need to change the source
- ◯ yes, it was done by the manufacturer service engineer
- ◯ yes, it was done by local staff with online support of the manufacturer
- ◯ yes but it was postponed
- ◯ yes, but it was cancelled and the source was not used
- ◯ Other (please enter text)

31. Did medical physicists in the department contribute to any COVID research initiatives?

- ☐ Help clinical teams in the design of databases
- ☐ Collection of data (eg CBCT)
- ☐ Radiomics studies
- ☐ Other (please enter text)

Lessons learned in radiotherapy physics during the Covid-19 pandemic

Morale and mental health

32. How did you keep the medical physics group united in these times? (tick all that apply)

- [ ] Email
- [ ] Texting groups (eg Whatsapp)
- [ ] Teleconferences (voice only)
- [ ] Video conferencing (with video)
- [ ] Unified communication and collaboration platform (eg Microsoft Teams group for videoconferencing, file share etc)
- [ ] Face-to-face meetings on site
- [ ] Other (please enter text)

33. What other actions have you/your colleagues initiated to handle the physical, mental and psychological impact of Covid-19?

Lessons learned in radiotherapy physics during the Covid-19 pandemic

Impact for the future

34. How has the pandemic changed the work culture in your department regarding working modes, communication and leadership? Tick all that apply

- [ ] Remote working becoming more acceptable
- [ ] Flexible working becoming more acceptable
- [ ] Using new online tools for tasks
- [ ] The team feeling more united
- [ ] The team feeling less united (ie divisions in the team)
- [ ] Enhanced trust in the leadership
- [ ] Less trust in the leadership
- [ ] Enhanced trust between the team members
- [ ] Less trust between the team members
- [ ] Other (please enter text)

35. Would you like any of the changes initiated to cope with the COVID 19 pandemic to continue afterwards in normal routine operation?

- ○ No
- ○ I don't know
- ○ Yes

36. If yes

- ☐ Working from home part of the time
- ☐ Automation of processes
- ☐ Streamlining patient-specific QA
- ☐ Hypofractionation
- ☐ Streamlining machine QA
- ☐ Other (please enter text)

37. Which ones are the most likely to remain?

- ☐ Working from home part of the time
- ☐ Hypofractionation
- ☐ Streamlining patient-specific QA
- ☐ I don't know
- ☐ Streamlining machine QA
- ☐ None
- ☐ Automation of processes
- ☐ Other (please specify)

38. Are you concerned about pressure to keep some changes made to cope with increased workload? (e.g. move of QA to evenings/ weekends)

- ○ No
- ○ Yes (please enter text)

39. Any other comment / experience you'd like to share?
