# Supplementary material for: Professional practice changes in radiotherapy physics during the COVID-19 pandemic
Source: Phys Imaging Radiat Oncol. 2021 Jun 22;19:25–32. doi: 10.1016/j.phro.2021.06.002 (PMC8216850; doi:10.1016/j.phro.2021.06.002)
Supplement: Supplementary Material A.II — Country Clustering. [file mmc3.docx]

**Supplementary material A.II: Country Clustering**

Countries were clustered using the analysis of Tenreiro Machado and Lopes [1] based on data from the European Centre for Disease Prevention and Control up to April 12 2020. Only countries with a new infection in the thirty days to April 12 were considered. Cluster analysis for the difference in cumulative number of infections (confirmed cases) as well as for the corresponding frequency domain (daily number of infection) were performed, the analysis in the frequency domain, i.e. based on the daily number of infection, was used. The authors used the Canberra distance to analyse the paired difference between countries which is more sensitive to proportional rather than absolute difference therefore mitigating the effect of population size.

| Supplementary table A.II | |  |
| --- | --- | --- |
| **Countries** | | **Number of responses** |
| **Cluster A (G^f^_11_)** | |  |
| France | | 27 |
| Germany | | 23 |
| Iran (Islamic Republic of) | | 1 |
| Italy | | 16 |
| Spain | | 36 |
| United Kingdom of Great Britain and Northern Ireland | | 62 |
| United States of America | | 57 |
| **Total Cluster G^f^_11_** | | **222** |
| **Cluster B (G^f^_12_)** | |  |
| Australia | | 22 |
| Austria | | 4 |
| Belgium | | 20 |
| Canada | | 11 |
| Chile | | 1 |
| Denmark | | 5 |
| India | | 20 |
| Ireland | | 5 |
| Netherlands | | 24 |
| Poland | | 1 |
| Portugal | | 4 |
| Republic of Korea | | 1 |
| Romania | | 3 |
| Russian Federation | | 25 |
| Serbia | | 1 |
| Sweden | | 4 |
| Switzerland | | 5 |
| **Total Cluster G^f^_12_** | | **156** |
| **Cluster C (G^f^_21_ and G^f^_22_ )** | |  |
| Bulgaria | | 2 |
| Egypt | | 1 |
| Estonia | | 1 |
| Finland | | 2 |
| Greece | | 2 |
| Hungary | | 2 |
| Malaysia | | 6 |
| *Continue on next page…*  *Table A.II. continued* | |  |
|  | |  |
| Malta | | 1 |
| New Zealand | | 9 |
| Norway | | 17 |
| Singapore | | 1 |
| Slovenia | | 1 |
| **Total Cluster G^f^_21_ and G^f^_22_** | | **45** |
|  | |  |
| **Not included in clustering analysis** | |  |
| Not included in [1] because no infection in the 30 days to April 12 | Bangladesh | 1 |
|  | Cuba | 1 |
|  | Cyprus | 1 |
|  | Nepal | 1 |
|  | Nigeria | 1 |
|  | Turkey | 2 |
| Missing from results in [1] | Brazil | 1 |
| No response in survey | Not defined | 2 |
