# Supplementary material for: Professional practice changes in radiotherapy physics during the COVID-19 pandemic
Source: Phys Imaging Radiat Oncol. 2021 Jun 22;19:25–32. doi: 10.1016/j.phro.2021.06.002 (PMC8216850; doi:10.1016/j.phro.2021.06.002)
Supplement: Supplementary Material A.III — Demographics by country cluster and overall. [file mmc4.docx]

**Supplementary material A.III: Demographics by country cluster and overall.**

| Supplementary table A.III: general demographics by country cluster and overall | | | | |
| --- | --- | --- | --- | --- |
| By cluster^1^ | A (N=222) | B (N=156) | C (N=45) | Overall  (N = 433) |
| **Gender (N (%)** | | | | |
| Male | 130 (59%) | 84 (54%) | 12 (27%) | 231 (53%) |
| Female | 89 (40%) | 71 (46%) | 30 (67%) | 194 (45%) |
| Non-binary | 0 | 1 (1%) | 0 | 1 (<1%) |
| Prefer not to say / no response | 3 (1%) | 0 | 3 (7%) | 7 (2%) |
| **Age group** | | | | |
| 18-24 | 0 | 2 (1%) | 0 | 2 (<1%) |
| 25-34 | 39 (18%) | 43 (28%) | 15 (33%) | 100 (23%) |
| 35-45 | 78 (35%) | 55 (35%) | 19 (42%) | 155 (36%) |
| 45-54 | 70 (32%) | 35 (22%) | 6 (13%) | 111 (26%) |
| 55-64 | 28 (13%) | 19 (12%) | 4 (9%) | 53 (12%) |
| 65+ | 5 (2%) | 11 (1%) | 1 (2%) | 8 (2%) |
| No response | 2 (1%) | 1(1%) | 0 | 4 (<1%) |
| **Position^2^** | | | | |
| Head of medical physics / management | 59 (27%) | 38 (24%) | 10 (22%) | 110 (25%) |
| Clinical medical physicist | 153 (69%) | 107 (69%) | 33 (73%) | 298 (69%) |
| Research / academic medical physicist | 6 (3%) | 7 (4%) | 1 (2%) | 15 (3%) |
| Other | 4 (2%) ^3^ | 4 (3%)^4^ | 0 | 8 (2%) |
| No response | 0 | 0 | 1 (2%) | 2 (<1%) |
| **Number of treatment units (linacs , brachy afterloader, proton units) in your department** | | | | |
| 1-2 | 48 (22%) | 43 (28%) | 5 (11%) | 98 (23%) |
| 3-6 | 104 (47%) | 65 (42%) | 22 (49%) | 198 (46%) |
| 7-10 | 49 (22%) | 22 (14%) | 11 (24%) | 82 (19%) |
| 10+ | 20 (9%) | 26 (17%) | 7 (16%) | 53 (12%) |
| No response | 1 | 0 | 0 | 2 (<1%) |
| **Number of patients treated per year** | | | | |
| < 2000 | 98 (44%) | 74 (47%) | 21 (47%) | 197 (45%) |
| 2000-4000 | 83 (37%) | 49 (31%) | 12 (27%) | 149 (34%) |
| 4000+ | 26 (12%) | 28 (18%) | 7 (16%) | 61 (4%) |
| No response | 15 (7%) | 5 (3%) | 5 (11%) | 26 (6%) |
| ^1^: 10 responses are not associated with any cluster (see appendix A.II.)  ^2^: see also analysis per profession group  ^3^: 2 trainees, 1 combined clinic/research, 1 principle research scientist primary standards  ^4^ :2 assistant professors, 1 trainee, 1 trainer and coordinator | | | | |
