# Supplementary material for: Professional practice changes in radiotherapy physics during the COVID-19 pandemic
Source: Phys Imaging Radiat Oncol. 2021 Jun 22;19:25–32. doi: 10.1016/j.phro.2021.06.002 (PMC8216850; doi:10.1016/j.phro.2021.06.002)
Supplement: Supplementary Material A.IV — Technical support availability and machine maintenance by country cluster and overall. [file mmc5.docx]

**Supplementary material A.IV: Technical support availability and machine maintenance by country cluster and overall.**

| Supplementary table A.IV: Technical support availability and machine maintenance by country cluster and overall. | | | | |
| --- | --- | --- | --- | --- |
| By cluster^a^ | A (N=214) | B (N=143) | C (N=44) | Overall (N=411) |
| **Was technical support for treatment unit available? (Q26)** | | | | |
| Yes | 128 (60%) | 95 (66%) | 30 (68%) | 261 (64%) |
| Yes, but only if linac could not treat | 67 (31%) | 29 (20%) | 8 (18%) | 106 (26%) |
| Yes, but only remote support | 15 (7%) | 18 (13%) | 1 (2%) | 34 (8%) |
| No | 0 | 2 (1%) | 1 (2%) | 3 (1%) |
| Other | 15 (7%) | 9 (6%) | 5 (11%) | 29 (7%) |
| **Did you have preventive maintenance done on a treatment unit during lockdown? (Q27)** | | | | |
| No, it was not planned anyway | 40 (19%) | 29 (20%) | 10 (23%) | 82 (20%) |
| No, it was planned but cancelled | 64 (30%) | 31 (22%) | 12 (27%) | 109 (27%) |
| Yes, it was planned and carried out | 90 (42%) | 65 (45%) | 16 (36%) | 176 (43%) |
| Other | 14 (7%) | 11 (8%) | 2 (5%) | 27 (7%) |
| No response | 6 (3%) | 7 (5%) | 4 (9%) | 17 (4%) |
| **Did you have to change the HDR/PDR source in your afterloader? (Q30)** | | | | |
| Yes, done by the manufacturer service engineer | 76 (36%) | 41 (29%) | 10 (23%) | 128 (31%) |
| Yes, done by local staff with online support of the manufacturer | 3 (1%) | 4 (3%) | 0 | 7 (2%) |
| Yes, but it was cancelled and the source was not used | 1 | 2 (1%) | 1 (2%) | 4 (1%) |
| Yes, but it was postponed | 7 (3%) | 7 (5%) | 6 (14%) | 20 (5%) |
| Other (see text) | 8 (4%) | 14 (10%) | 4 (9%) | 27 (7%) |
| No, we have a HDR/PDR afterloader but did not need to change the source | 52 (24%) | 29 (20%) | 11 (25%) | 97 (24%) |
| No, we don't have a HDR/PDR afterloader | 58 (27%) | 37 (26%) | 7 (16%) | 105 (26%) |
| no response | 9 (4%) | 9 (6%) | 5 (11%) | 23 (6%) |
| ^a^: 10 responses are not associated with any cluster (see supplementary material A.II)  Acronyms: HDR: high dose rate, PDR: pulse dose rate. | | | | |
