# Supplementary material for: Professional practice changes in radiotherapy physics during the COVID-19 pandemic
Source: Phys Imaging Radiat Oncol. 2021 Jun 22;19:25–32. doi: 10.1016/j.phro.2021.06.002 (PMC8216850; doi:10.1016/j.phro.2021.06.002)
Supplement: Supplementary Material A.V — Free text comments from the respondents. [file mmc6.pdf]

Supplementary material A.V: Free text comments from the respondents

Here we present the unedited comments from the respondents (anonymized) grouped by theme.

Positive experiences / proud of their team and leadership

- We adapted well.
- I think our centre has coped very well in the pandemic and has made changes that have benefitted everyone. The only downside occasionally has been communication between each other and between different staff groups. But on the whole, I am very proud to have worked in this team and to have overcome different challenges
- I am proud of how my department adapted and worked together. Many things we implemented will be beneficial and remain. We face a busy workload soon, as patients that were delayed start to come back, and with 2 extra pressures of commissioning a linac, and implementing SABR. I am confident we will be able to pull through.
- The department has coped very well with staff very willing to work differently for a while. We will review opinions as to what works well in time as shift patterns introduced might suit some not others. Degrees of homeworking have certainly been positive for some. IT infrastructure to support remote working was not in place initially and the positive is that covid-19 has pushed many changes to getting remote access that may benefit the service in the future e.g remote mark-up, planning, and perhaps soon sign off, as well as homeworking which would otherwise taken forever to get IT depts. to implement. We unfortunately are not paperless and it would have been very easy to move all work off site if we had been.

Negative experiences / disappointed in leadership

- I'm personally disappointed in leadership and how they fanned flames of fear and division instead of trying to be thoughtful and help employees navigate change
- The leadership in my department was terrible. They were below average before hand. They failed the test.
- Our administration's main concern was to track productivity of staff moving to remote work. The decline in patients and treatments were more of a concern than staff safety.
- Very angry and disappointed at the way we completely carried on as normal in our Physics department, but without any cleaning products to clean our work surfaces, phones or keyboards, as they were needed in the clinical areas. From talking to colleagues in other centres I know this is not normal. It definitely damaged morale.
- Radiotherapy management appeared to think they were superior to physics during this pandemic. Our thoughts, feelings were no longer equal to theirs. It felt as if the radiotherapy managers and superintendents thought were less deserving of credit and gratitude than them.

- Very significant changes to the management structure in our department have been rushed through under the cover of the COVID crisis without due scrutiny or consultation.

Physical/mental health and well-being, community

- Physical fitness of Physicists is very very important to cope with the Pandemic.
- Concerning about each other health is good to kept on!
- the psychological effects have been profound and very difficult to deal with. Pressures have been not well understood and nothing has been put in place to help
- As always, I am impressed but not surprised at the ESTRO MP community. There was a lot of sharing of experience across the countries and I received and sent many emails and Whatsapp texts to colleagues to check in with each other and to share. It is very helpful to have access to such a community!

Working at home and efficiency

- Working from home has been surprisingly efficient. It has also made it possible to avoid public transport. However, the workload and stress has increased for those remaining on site. It has created several conflicts.
- Disappointing the hospital management decided not to implement working from home as an option. Many people could have easily worked from home with little change in practices. We were told not to work from home! Remote access to systems what not granted, even though the infrastructure was available.
- My wish is that remote work/flexibility of work schedule continues when and if this pandemic ends. The balance lies in being present in the clinic (face time) while having the opportunity to work remotely. Most of my colleagues found that they were more productive when working from home. Historically physics staff has been the catch all for clinic issues (IT related, linac troubleshooting etc) which has added value to our roles but can also lend to constant interruptions. Hopefully going forward we can find a suitable normal.
- partly working from home should be encouraged. Hospital direction and head of physics should become convinced that it is possible.
- We go on working at home once a week, and we would like maintain forever
- Whilst working at home was available, it is often less productive as systems are slower and reliance on personal IT equipment to run them.
- There is a disparity in workload when working from home and when coming to work. Working from home has addressed some work/life balance, but we pay for it on the weeks we are in work!

Call for learning from specific aspects

Technical aspects, IT security and machine troubleshooting:

- Remote engineering support only works if the infrastructure is there to support it. A searchable database of previous faults/errors is a must. An easy and fast way for machine operators to note warnings and faults that can be monitored by engineering remotely makes a huge impact on their ability to prevent machine down time. The availabilty of engineers to answer calls from runup until after the machine is shut down is critical.
- IT infrastructure and the passing of patient data between work and home is a risk
- Hope we will learn from our weakness during the pandemic like allowing every physicist to have a performant laptop with all the applications we daily use

Dealing with infectious/at risk patients

- With reduced staffing moving patients became a task that involved all staff including physics and dosimetry. I think it would go over better if we all learned safety precautions when dealing with infectious patients.
- We are now taking extra precautions for patients who come from old folks homes in order not to infect them and those who share the same roof.
- I wonder if we'll be expected to use PPE as a new norm. Might be a good idea to wear masks whenever possible... but I really hate them.
- This has forced us to become more aware of universal precautions.
- it is a good thing to mantain also the protective clothes and cleaning methods in the future at the linacs and diagnostic rooms.

Other topics to learn from

- cross training is more important than ever
- Radiation fear is less important now than life threatening diseases like COVID-19. People fear radiation less than before, and that's good. People has just started to think in terms of risk and probability, and this is the main lesson for human kind for our own survival. At the end "black swans" are unexpected, but this was not a "black swan" and it was completely preventable. People should not rely that much on governments policies, that take too much time and consensus, and start caring for ourselves start taking actions right now. As Nassim Nicholas Taleb put it: "Survival is everything". See you in Madrid, before COVID-21.
- I think it’s important that when Covid is under control we don’t simply move back to how things were. A lot of the changes forced by Covid have been positive for staff and the hospital and we should make sure we keep them.
- We need a clear headed debate, over time, about what changes to keep and what to discard.

Concerns about the future workload

- We expect to experience an increased workload later this year as patients were delayed for surgery or put on other therapies first. also, population screening for

breast and colon cancer were interrupted, which might result in higher stages at diagnosis

- In the last 2months decreased the number of rt treated patients by 35-40%, and it's not because of erradication of cancer.....

Research and project development

- For those who have an academic component (research and teaching) in their responsibilities, Covid-19 seems to have had a significant impact. Research productivity is down considerably depending on the nature of the research and much of the teaching is converted to on-line teaching. There are significant limitations for medical physics residents to perform their 'hands-on' activities.
- During these months we had "maximum" staff. No conferences, travelling etc. Several projects which were on the pipeline could be done. A lesson learned is that focused work on projects has worked better than for instance one day a week.   The most important lesson learned is the confirmed importance of av very good cooperation between physicists, engineers and the medical unit in radiation therapy which has allowed to work in a constructive and effective way on the specific Covid routines.
- Routine work can continue without face-to-face contacts, but project develepomentts strongly requires direct/informal contacts (at coffe machine, hearing a phone call between 2 other colleagues, just after a meeting, ...)

Other precisions on personal/local situation

- The division of the team was far from complete, there was a lot of overlap between the teams.
- Due to national lock down early on in the crisis in New Zealand the medical physics group was largely unaffected by the COVID-19 emergency. The treatment RTs were affected more due to the increased risk. Our standard QA schedules already minimized overlap with treatment, all being at the end of a day.
- For the most part it has been business as usual in my clinic.
- We were lucky in that we escaped the high numbers of cases else where but hospital management relucatnce to authorise working from home/different work patterns etc made many of the solutions other centres found to maintaining a MedPhys Provision impossible. I had grave concerns that if one member of the team went down we would all have to isolate and service continuity could not be maintained.
- RTs worked in designated pairs on machines and had to block out tea breaks. Extended day even further.   State border closures added increase pressure to department (border town) as patient taking anywhere from 10-70 minutes to get through border checks.
- Team members with mild illness symptoms have are now more inclined to work from home rather than come into work. During the 'lock down' phase I worked from home for 7 weeks due to 'pre-existing a health condition. there were 2 teams working week

about. The work load of my routine QA shifted to those on site. There was an increase in out of hours work for those on site, but it was managed well.

- Delayed replacement of equipment due to vendor staff not being able to travel has forced us to delay replacement of linac and Aria upgrade which impacts department efficiency and has created problems for planning of other major projects to be finalized during remainder of financial year
- some public hollidays could be used for yearly QA and if not shift work especially late shift could be splitted to perform other QA stuff,
- we had a decreasing in the number of patients
- I did move some of my QA to weekends, but shifted back to QA during normal days of the week due to a decreased patient load on some of the machines
- We have changed very little in physicist routine. We have two full time physicists. Only our dosimetrist is working from home, about 2/3 of her days. We are currently looking to hire a second part-time dosimetrist.
- We already hypofractionated many patients before Covid-19. So, impact on hypofractionation due to the crisis is low in our clinic.
- We drastically introduced hypofractionation (eg Fast Forward protocol for breast treatment), postponed less urgent treatment (eg prostate) and postponed projects to create free time slots and oxigen in case machines needed to be shutdown if staff would be reduced when the virus should hit the team (RTT, RadOnc and/or MPE), fortunately, this didn't happen and we had a remarkable quiet period.
- We moved to extended hours due to trying to isolate our therapy team staff. It was very difficult to balance isolation of staff and also preserving number of hours for staff so as to not cut hours. Additionally, patient volume fluctuated widely between low and high number of patients as the physicians decided to hold off on treating some patients, and then a large number of patients needing to be rescheduled once the pandemic slowed down. Difficult to manage.
- Physics staff put massive effort into very quickly rewriting clinical protocols for new hypofractionated treatments. Also had to flexibly move to 6-day treatments for head and neck patients (DAHNACA) for patients who couldn't have normal chemo.
- There were absolutely no Changes for physicists.
- For those of us working not in home office it was... business as usual.
- Communication between two staff group sometimes wasn't enough precisize
- I couldn't find much difference in terms of machine QA. In india physicists work on Saturdays for QA. However, patient specific QAs were carried out on Saturdays. That was pretty hectic
- Sweden got it's first COVID patients relatively late, so we had a time to prepare our clinic for changes to come. some changes that were just downprioritated lately had a chance to be introduced in the Clinical practice
- I would have appreciated more official "rules" or guidelines to follow from estro or dmpg to present our head of department
- How could we have helped as medical physicists in COVID-19 front line research and clinical practice?

- Do not go with the mass. Evaluate your own process and make it work for your department. Be flexible where it is possible and strict where it is needed. Make decisions preferably earlier than later.
